# Supplementary material for: Comparative Study of Antioxidant Potential of Selected Dietary Vitamins; Computational Insights
Source: Molecules. 2019 Apr 26;24(9):1646. doi: 10.3390/molecules24091646 (PMC6540138; doi:10.3390/molecules24091646)
Supplement: Supplementary file 1 [file molecules-24-01646-s001.pdf]

Table 1. Reaction enthalpies in kJ mol^{-1} in water for the selected dietary vitamins, employing functional of B3LYP, M05-2X and M06-2X

Vitamins	BDE	IP	PDE	PA	ETE
Vitamin A					
B3LYP	403	620	1114	273	316
M05-2X	355	646	1098	276	257
M06-2X	357	650	1090	277	254
Vitamin B1					
B3LYP	419	1020	649	250	354
M05-2X	435	1124	626	255	358
M06-2X	435	1127	624	255	355
Vitamin B3					
B3LYP	441	892	869	149	478
M05-2X	482	919	873	142	518
M06-2X	481	915	874	146	510
Vitamin B6					
B3LYP	340	794	866	182	344
M05-2X	359	817	839	179	358
M06-2X	362	816	838	182	355
Vitamin C					
B3LYP	301	797	807	125	362
M05-2X	319	813	800	121	376
M06-2X	318	809	801	125	368

6-311G** basis set was used for all calculations
